# Cavernous hemangioma of the cisternal segment of the auditory nerve: case report

**DOI:** 10.1186/s12883-023-03275-7

**Published:** 2023-06-06

**Authors:** Zuan Yu, Tingming Lu, Tao Yu, Risheng Liang

**Affiliations:** 1 Department of Neurosurgery, The First Affiliated Hospital of Wannan Medical College (Yijishan Hospital of Wannan Medical College), Wuhu, Anhui Province China; 2 Department of Neurosurgery, Fujian Medical University Union Hospital, Fuzhou, Fujian Province China; 3 The Translational Research Institute for Neurological Disorders of Wannan Medical College, Wuhu, Anhui Province China

**Keywords:** Cisternae of the cranial nerve, Cavernous hemangioma, Cistern-type

## Abstract

**Background:**

Extraaxial cerebellopontine angle cavernous hemangiomas are rare and their diagnosis and treatment are challenging.

**Case presentation:**

A 43-year-old female was admitted to the hospital who had repeated hearing loss in her left ear accompanied by tinnitus. Magnetic resonance imaging revealed a hemangioma-like lesion in the left cerebellopontine angle extra-axial cisternal segment. During the surgery, it was found that the lesion was located in the cisternal segment of the root of the auditory nerve. Postoperative pathological examination confirmed that the lesion was a cavernous hemangioma.

**Conclusion:**

We report a case of cavernous hemangioma in the brain spatula cisternal segment of the left auditory nerve. For cranial nerve CMs early diagnosis and surgical removal may maximize the chance of a positive outcome.

## Introduction

Cavernous angiomas (CMs) are benign vascular malformations that account for 10–15% of all vascular malformations of the central nervous system [1, 2]. Extra-axial CMs are exceedingly rare and occur mainly in the floor of the middle cranial fossa, especially in the cavernous sinus [3–5].

CMs of the cerebellopontine angle (CPA)can be classified according to their site of origin. CMs of the internal auditory canal (IAC) can be categorized as the IAC type, and CMs arising in the cisternae of the cranial nerves (CN) can be categorized as the cisternal type [6]. The IAC type develops from the dura or CN, whereas the cisternal type develops exclusively from the vascular plexus of the CN [6]. Extra-axial cavernomas located in the CPA have been rarely reported in the published literature, moreover, and those located at the cisternae of the CNs are even rarer. We report an extra-axial CM attached to the cisternal segment of the auditory nerve.

## Case presentation

**Fig. 1:**
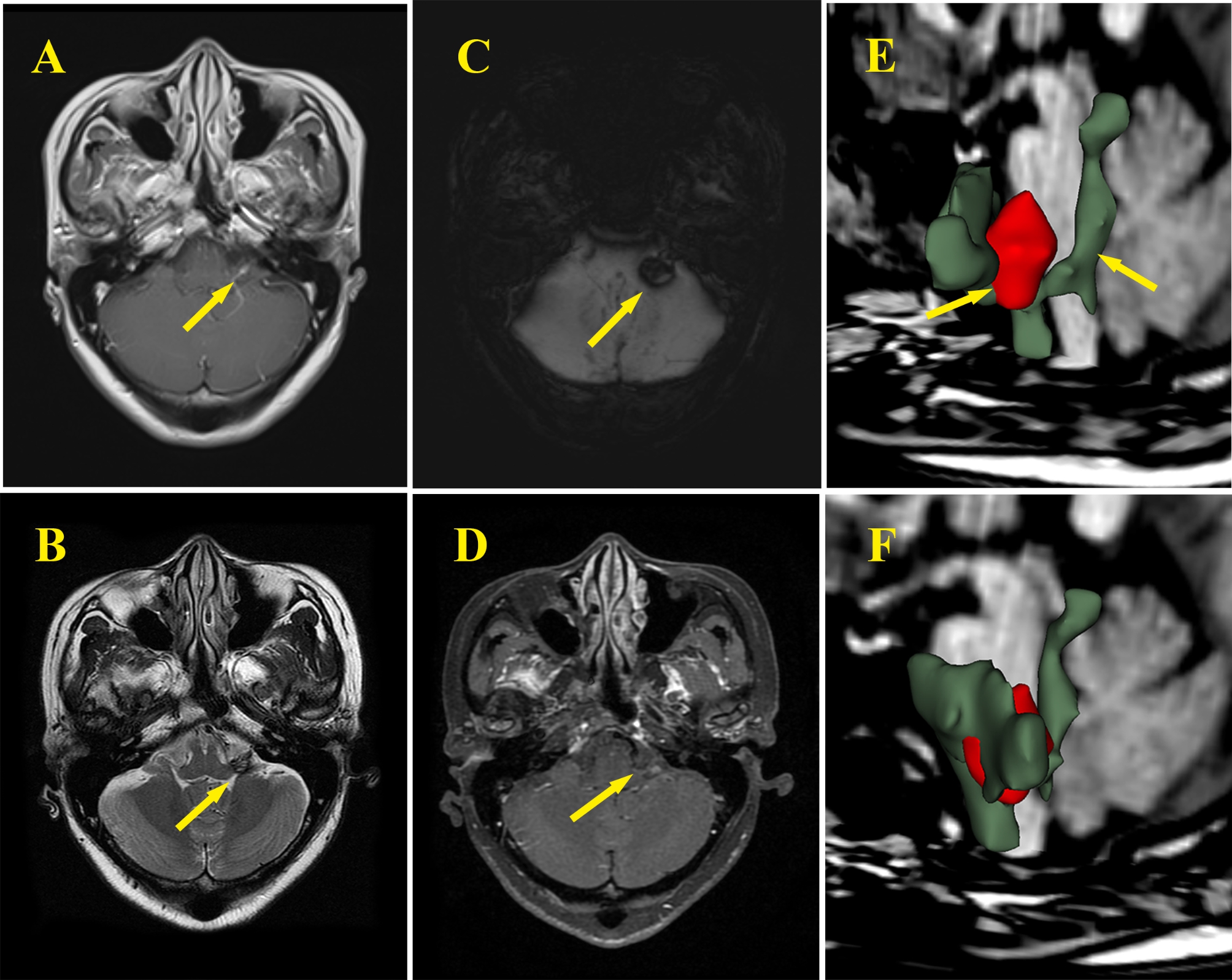
The MR image showed the lesion of the cisternal segment of the root of the left auditory nerve **(A, B, C, D)**. The reconstruction using 3D-slicer software shows the anatomical relationship between the lesion (red) and the cisterna (green) **(E, F)**

A 43-year-old female presented with hearing loss in the left ear and tinnitus for > 1 month. The patient’s condition did not improve significantly after the administration of dexamethasone, and she required referral to our center. The patient’s history revealed a left-sided hearing loss that self-resolved after a period of rest 10 years prior. She had no specific medical or family history of cerebrovascular disease. Audiological function tests showed sensorineural hearing loss, and there was no abnormality in facial nerve function. The lesion in the left CPA area showed isointensity on T1-weighted imaging and mixed hyper and hypointensity on T2- weighted imaging images, and it was not significantly enhanced after contrast administration. It presented as hypointensive signals and hyperintensive signals, which were evident on the margins of lesions on susceptibility weighted images (Fig. 1). Cerebral angiogram showed no evidence of cerebral aneurysm or arteriovenous malformation.

**Fig. 2:**
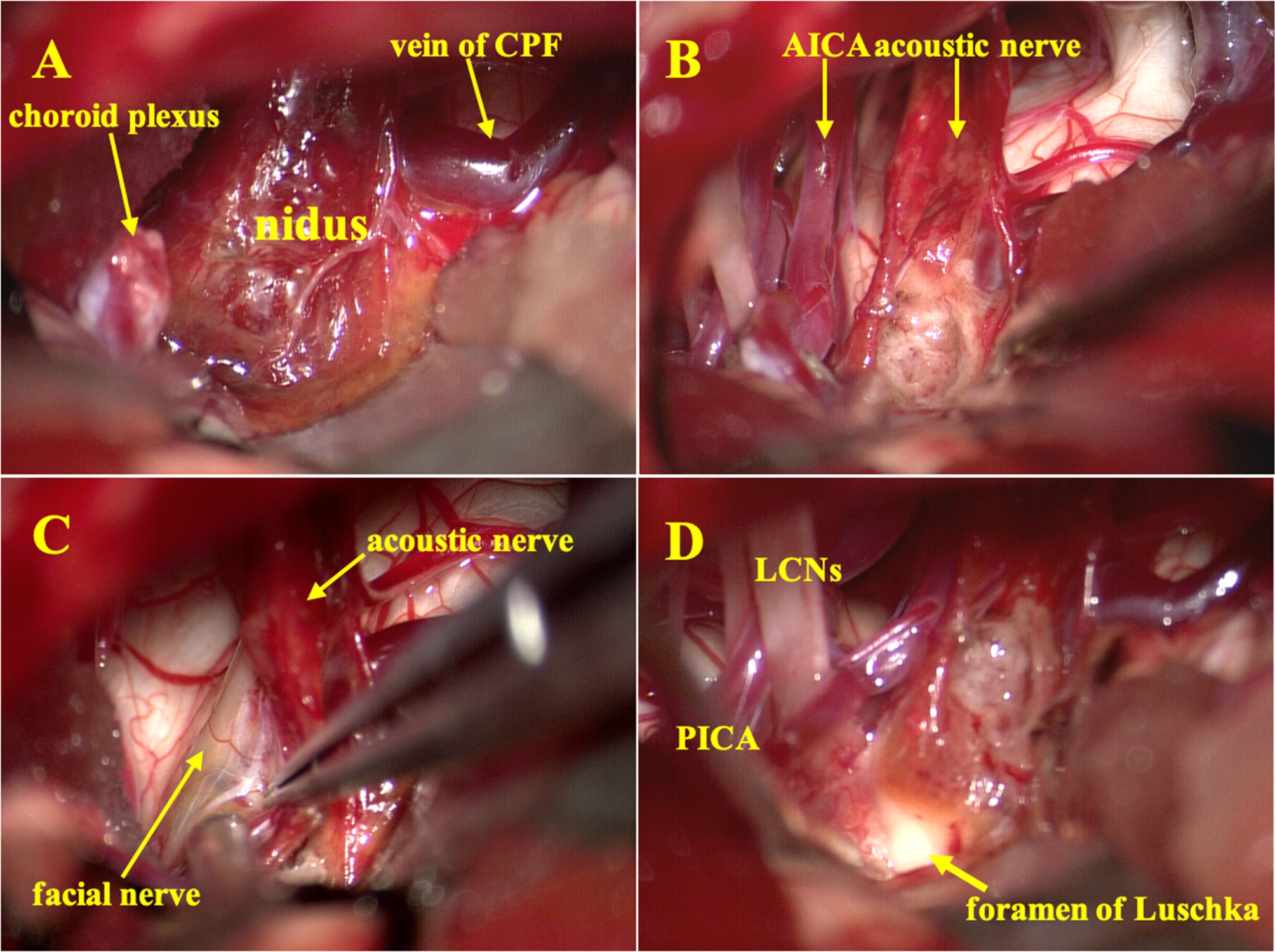
Intraoperative pictures of the left auditory nerve near the brainstem before and after resection. **(A)** After microdissection and lifting of the flocculus, the lesion was exposed. **(B)** The location of the lesion and its relationship with the surrounding neurovascular structure became obvious after the lesion was completely resected. **(C)** The cochlear nerve was gently pulled upward to expose the proximal end of the facial nerve. **(D)** Relationship between the innermost side of the lesion and the lower cranial nerves and the foramen of Luschka

After extensive discussions with the patient about various surgical options and risk benefits, microsurgery was performed to remove to the lesion, and the surgery used a left retrosigmoid approach, with the patient in the lateral position under neuromonitoring. The surgeon used the brain spatula reasonably and intermittently, combined with dynamic micro traction technology to explore and treat the lesion in the CPA area. The lesion was dark red and comprised a large number of tortuous small thin-walled vessels mixed with old bleeding, It was well-margined with dimensions of 1.0 × 0.8 × 0.5 cm and was buried in the proximal cisternal portion of the auditory nerve. The angioma was successfully resected en bloc, and the microvessels feeding it were cauterized with 3 w, which was a weak power of electrocoagulation for hemostasis, so as to ensure that no heat was transmitted to the facial nerve. The bulk of the lesion appeared to be located on the vestibular nerve with relatively minor involvement of the cochlear nerve structures whilst sparing the facial nerve. Although the veins of the cerebellopontine fissure adjacent to the lesion were thicker, there was a glial layer between them, and no vascular branches were connected. This cerebellopontine fissure vein did not appear to be the tortuous vein seen in the preoperative digital subtraction angiography (Fig. 2).

**Fig. 3:**
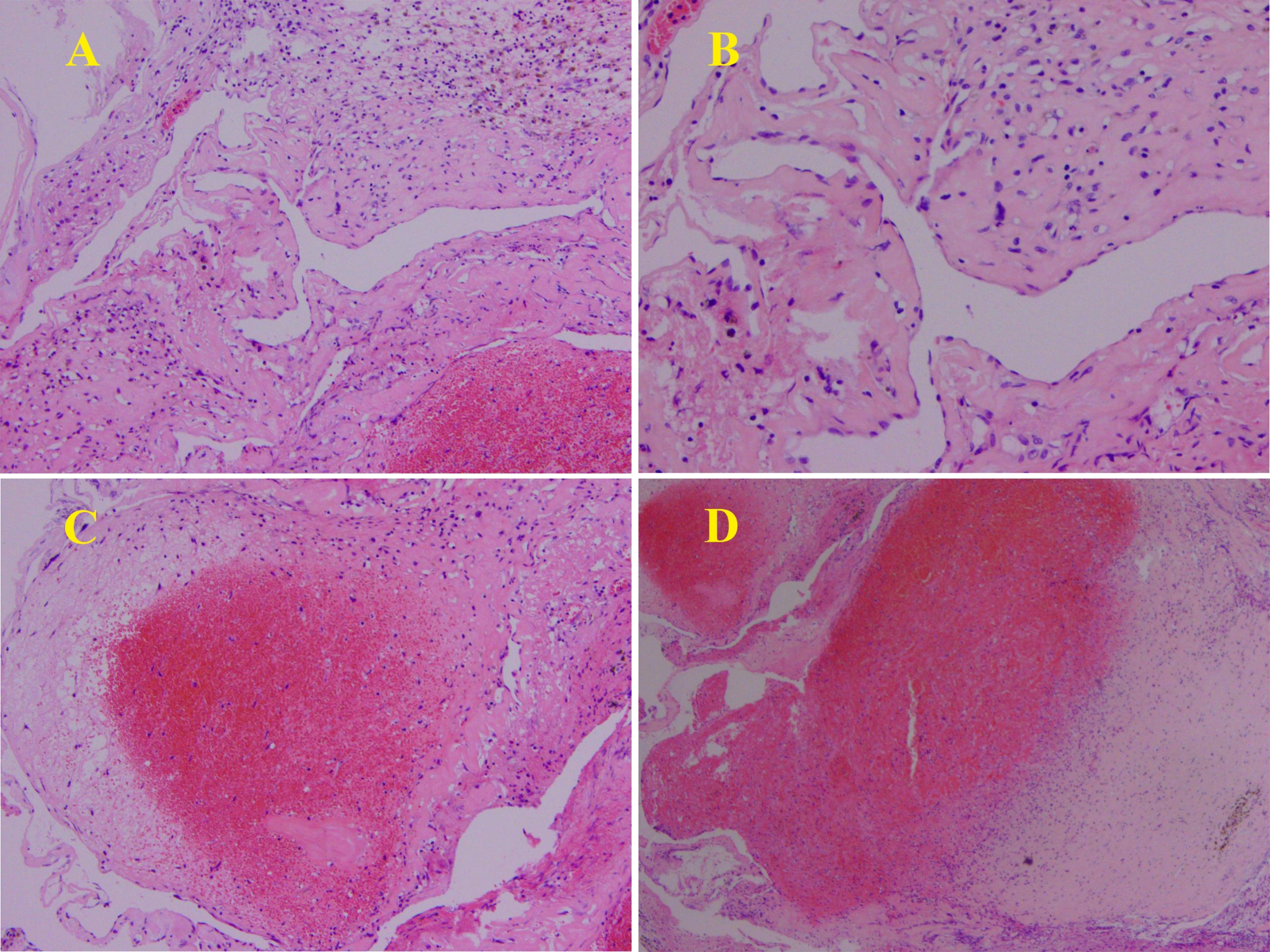
Microscopically, it showed an irregularly thin-walled dilated blood vessels lined with endothelial cells (**A** (40 x), **B** (100 x)).The lumen is filled with blood, and some of the partially vascular lumina are thrombosed (**C** (40 x), **D** (100 x))

**Fig. 4:**
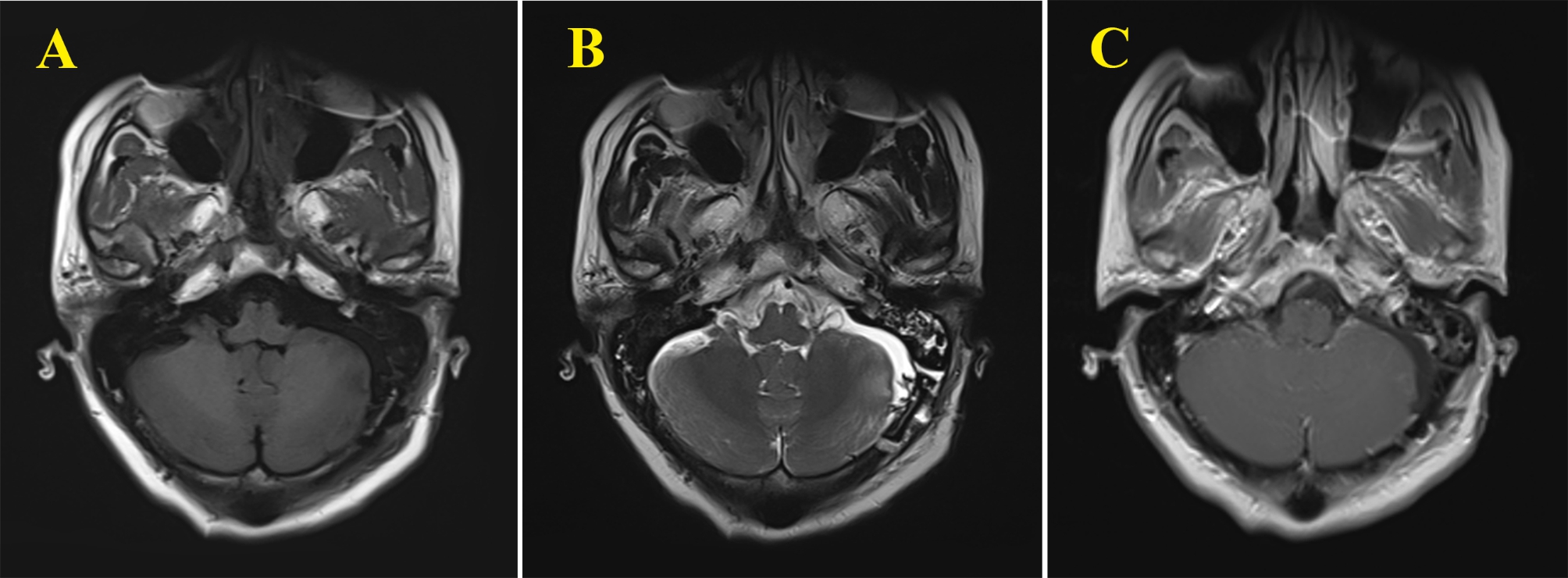
Postoperative MRI showed that the lesion had been completely removed (**A, B, C**)

The pathology was reported to be a cavernous angioma with thrombosis. Immunohistochemical staining for CD31, CD34 and ERG were positive within luminal endothelial cells. Immunostaining for D2-40 was negative (Fig. 3). Postoperatively, the patient’s hearing was decreased as before, but there was no facial paralysis. Reexamination of head magnetic resonance imaging (MRI) showed that the lesion had been completely removed (Fig. 4). The postoperative disease course was uneventful, and the patient was discharged.

## Discussion

CMs of CNs are extremely rare, leading to their diagnosis and treatment being challenging. Ferrante L et al. reported the first case of CM in the cisternal segment of the eighth cranial nerve near the brainstem in 1998, which was associated with venous aneurysm [7]. To the best of our knowledge, ours is the second case reported to date. Based on the intraoperative observation, the lesion in our case does not appear to be accompanied by a venous anomaly (Fig. 4).

Owing to the narrow space of the CPA and the complex anatomical structure surrounding it, it is challenging to completely remove the cranial nerve cisternal segment CM, however, this surgery can be achieved under the current conditions of microscopic technology and electrophysiological monitoring [8]. However, preservation of auditory and facial nerve(especially the former) function after surgery is still a difficult problem. It has been reported that hearing retention is difficult to achieve in patients with CMs located in the CPA [9, 10], This difficulty may be related to the more fragile and less ischemic tolerance traits of the auditory nerve, and regional deposition of hemosiderin and severe adhesion after repeated bleeding. Comparatively, the functional recovery of the facial nerve reported in the literature seem to be better. Because these CMs may less likely be derived from CN VII but only compress or slightly adhere to the facial nerve [11–13].

We agree that preservation of nerve function after surgery mainly depends on the lesion origin. Lesion hemorrhage and reduced blood supply to the nerve during growth lead to nerve dysfunction before surgery [14]. Furthermore, it is our belief that the factors affecting the recovery of CN function also include the existence of an arachnoid interface between the nidus and CN, a closer the adhesion between the nidus and CN, a higher the degree of infiltration, and a lower the integrity of the arachnoid interface, leading to greater damage to the nerve and a lower probability of CN functional recovery when completely resected. During surgery in this case, we found that the lesion mainly involved the vestibular nerve. Despite a decrease in the patient’s hearing postoperation, her facial nerve function was intact after surgery.

On the basis of the abovementioned findings, early diagnosis is crucial for CMs of the CN, which can result in earlier complete resection of the lesion and anatomical and functional preservation of the nerve. MRI is the preferred examination for the identification and follow-up of CMs located in the cisternal segment of the CN. Some studies have noted out that early off-axis CM and CN have colloidal gaps that are prone to plane formation, which facilitates surgery [10, 15]. CMs close to the brainstem have a poorer outcome and higher risk of bleeding and rebleeding [16]. In our case, the patient suddenly developed hearing loss 10 years ago, which recovered naturally. It can be speculated that the lesion may have already existed at that time. Therefore, although rare, radiologists and neurosurgeons should be aware of the possibility of CM in the cisternal segment of the CN. Early diagnosis and surgical intervention of this particular type of CM can reduce the difficulty of operation while also preserving as much neurological function as possible.

## Conclusion

Herein, we report a case of cavernous hemangioma in the proximal cisternal segment of the root of the left auditory nerve. It is plausible that early diagnosis and surgical intervention for CMs originating from cranial nerves could result in favorable nerve function.

## Data Availability

Data available from the corresponding author upon reasonable request.

## Abbreviations

CM: Cavernous malformation
CN: Cranial nerve
CPA: Cerebellopontine angle
MRI: Magnetic resonance imaging
IAC: Internal auditory canal
CN VII: Facial nerve
CN VIII: Auditory nerve
